# Normalization and missing value imputation for label-free LC-MS analysis

**DOI:** 10.1186/1471-2105-13-S16-S5

**Published:** 2012-11-05

**Authors:** Yuliya V Karpievitch, Alan R Dabney, Richard D Smith

**Affiliations:** 1 School of Mathematics and Physics, University of Tasmania, Hobart, Tasmania, Australia; 2 Department of Statistics, Texas A&M University, College Station, TX, USA; 3 Fundamental and Computational Sciences Directorate, Pacific Northwest National Laboratory, Richland, WA, USA

## Abstract

Shotgun proteomic data are affected by a variety of known and unknown systematic biases as well as high proportions of missing values. Typically, normalization is performed in an attempt to remove systematic biases from the data before statistical inference, sometimes followed by missing value imputation to obtain a complete matrix of intensities. Here we discuss several approaches to normalization and dealing with missing values, some initially developed for microarray data and some developed specifically for mass spectrometry-based data.

## Background

High-throughput mass spectrometry (MS) has become an important technology for protein identification and quantitation due to its ability to rapidly provide identification and quantitation of thousands of peptides [1]. In this manuscript we focus on the studies where proteins are first digested into constitutive peptides by means of chemical or enzymatic digestion. Mixtures of peptides from complex biological samples are generally subjected to liquid chromatography (LC) to separate the peptides in time such that the mass spectrometer is provided with only a small portion of all peptides in a sample (based on some physiochemical properties) at a given time. Separation allows for more peptides to be detected by the mass spectrometer. The liquid coming from the LC separation column is generally electrosprayed to form molecular ions that are then subjected to MS or MS/MS analysis. The resulting mass spectra are compared to the theoretical or previously observed peptides to produce peptide identifications and subsequently quantitation of those peptides (and often the parent proteins) [2-5].

Systematic bias is inherent in MS-based data due to complex biological, experimental and technical processing. Bias, which may be loosely defined as any non-biological signal, may occur due to many factors, including variations in sample processing conditions, instrument calibrations, LC columns, changes in temperature over the course of an experiment, etc. One may observe systematic biases in mass measurement accuracy, LC retention times, and/or peak intensities. In an effort to better enable comparisons between samples, it is desirable to remove any excess technical variability by utilizing various normalization techniques. For example, LC-MS samples may be aligned in terms of their LC retention time and mass profiles, or nonlinear modeling may be employed to capture systematic errors in mass measurements [6] or peak intensities [7-9]. Several normalization methods have been imported from microarray studies such as *central tendency*, *lowess *regression and *quantile *normalization [10,11]. Other normalization methods were developed specifically for MS-based proteomic data [8,9].

Another challenge in quantitative proteomics is wide-spread missing data (i.e. missing identifications or abundance values). A peptide intensity value may be missing due to several mechanisms, including: (i) the peptide truly is present at an abundance the instrument should be able to detect, but is not detected or is incorrectly identified, (ii) the peptide truly is present but at an abundance below the instrument's detection limits, and (iii) the peptide is not present. Different methods for dealing with missing values should be used depending on the mechanism that gave rise to a missing value. In case (i), using observed values to impute missing values or simply ignoring a few missing values is appropriate. However in cases (ii) and (iii), when a peptide abundance is below our ability to detect it, simple imputations based on observed values are not appropriate [12]. Such values are said to be *censored*; ignoring or imputing censored values based on the observed data will overestimate peptide abundances and lead to biased results.

Analysis of MS data almost always involves dealing with both bias and missing values. Deciding which normalization to use and when can be challenging. For example, one needs to make a decision on whether to impute the missing values first and normalize next, or the other way around. All work is generally performed on the logarithm (log) scale of the abundances/intensities. This simplifies statistics that follow, as log abundances are often approximately normally distributed. Logarithm base two is preferred for the ease of fold change interpretation but any logarithmic transformation will produce approximately Normally distributed intensities. Here we use terms abundance and intensity interchangeably.

## Methods

### Normalization

In the context of -Omics applications, bias can generally be defined as non-biological signal; that is, systematic features of the data that are entirely attributable to experimental or technical aspects. There are many sources of bias in LC-MS data, all of which have the potential to affect the measured peptide/protein expression levels (e.g., non-optimal ionization efficiencies in complex samples, differences in LC columns, differences in sample preparation and data acquisition between technicians). The term normalization refers to the process of removing such biases. There are many different approaches, but we focus our discussion on those that are most widely used or have properties that work especially well for proteomic data.

A *global adjustment *is often used to force the distribution of the log intensity values to center around a constant such as mean, median or some fixed value for each sample [7]. Here an assumption is made that most peptide abundances do not change, so the distribution of intensities across different samples should be similar. A constant may also be based on a subset of peptides coming from known conserved house-keeping proteins [13]. Global adjustment can correct for differences in the amounts of material loaded for each sample, but cannot capture more complex (e.g., non-linear and intensity-dependent) biases.

Robust scatter plot smoothing or *lowess *regression is another widely-used normalization technique adapted from the microarray setting [7,11]. Scatterplot smoothing techniques work with so-called MA plots ("minus vs. average" for comparing the intensities of two samples). These techniques were developed in the context of two-color gene expression microarray studies, where two fluorescent dyes are used on each array. While there is a natural (internal) reference in two-color microarrays, in proteomic studies a reference sample must be selected against which all the other samples will be normalized. Selection of such reference sample is usually arbitrary. Lowess performs local linear fits dependent on the user-defined fraction of points to be used for smoothing at each point. The fraction value is mostly arbitrary and suboptimal value may reduce the efficiency of the normalization and result in poorly normalized data. Empirically selected value of 0.4 has been used in several studies [7,10]. Berger et al. proposed an optimization-based procedure for estimating the fraction value [14]. Scatterplot-smoothing techniques are able to capture non-linear intensity-dependent biases and are therefore more flexible than global adjustments.

ANOVA and regression models can effectively estimate and remove systematic biases when sources of bias are known exactly [15]. Consider an LC-MS experiment in which protein- and peptide-level identifications are obtained for samples from two or treatment groups, with the samples run in two or more batches. A sensible model would be:

(1) $$y_{ijkbl}=\text{Pro}{\text{t}}_{i}+\text{Pe}{\text{p}}_{ij}+\text{Trea}{\text{t}}_{ik}+\text{Batc}{\text{h}}_{ib}+\varepsilon_{ijkbl}$$

Where *y_ijkbl _*is the log-transformed peak abundance for protein *i*, peptide *j*, comparison group *k *and batch *b*; Prot*_i _*is the overall mean intensity for protein *i*; Pep*_ij _*is the offset for peptide *j *from *i*th protein mean; Treat*_ik _*and Batch*_ib _*are mean differences between treatment groups and batches, respectively; and the *ε *term represents random error. For a given protein, the peptide effects are constrained to sum to zero; that is, $\Sigma_{j}\;\text{Pe}{\text{p}}_{ij}=0$. Similarly, for a given protein, the treatment effects are constrained to sum to zero; $\Sigma_{k}\;\text{Trea}{\text{t}}_{ik}=0$ and so do batch effect, $\Sigma_{b}\;\text{Batc}{\text{h}}_{ib}=0$. The $\varepsilon_{ijkbl}$ term represents random error, and follows the Normal distribution with mean zero and variance $\sigma_{ij}^{2}$ assuming a separate error variance for each peptide but a common variance for each treatment group and batch in the same peptide. The model above can estimate and eliminate bias attributable to batch effect, and other terms can be added to the model to estimate other known sources of variation. In the above model batch effect would capture any variation due to samples processed in one group or close proximity in time. An alternative model for abundance would replace our peptide fixed effects with random effect, resulting in a mixed effects model [16]. In cases when technical replicates are obtained subject random effects also can be introduced. The qualitative difference between the fixed and mixed effects models would be minimal. However, it will not generally be possible to identify all of the relevant sources of bias to sufficiently model biases with ANOVA. Furthermore, biases due to batch effects, say, are likely to be more complex than simple constant shifts, like those specified in model (1). More realistically, biases would be modeled via flexible nonlinear functions, but this complicates the analysis [17]; see Supervised Normalization of Microarrays (SNM) for a slick solution in the microarray setting [18]. Finally, the use of peptide-specific models for preprocessing may overfit the data and invalidate downstream statistical inference [19].

*EigenMS*, an adaptation of surrogate variable analysis for microarrays [17], was developed specifically for MS data [8]. *EigenMS *uses singular value decomposition (SVD) on model residuals to identify trends attributable to bias. This allows for bias of arbitrary complexity to be captured as 'eigenpeptides' and subsequently removed. This means that the researchers do not need to know sources of bias to be able to remove them. *EigenMS *has several beneficial features for proteomics data normalization. First, it is applicable to data with widespread missing values, as is common in MS-based proteomics. Second, the *EigenMS *algorithm is well-suited for inclusion in an existing proteomics analysis pipeline, as it does not require any special downstream steps or housekeeping. Automated selection of the number of significant bias eigentrends simplifies the normalization process, which can be applied to identified peptide abundances or abundances of unidentified features.

When doing differential expression analysis it is advised to check the distribution of the p-values as a diagnostic plot. Figure 3 (left panel) shows the distribution of p-values under the null hypothesis, i.e. no differential expression. As expected the p-values are approximately uniformly distributed on the [0 1] interval [20,21]. Figure 3 (right panel) shows skewed distribution of p-values which may indicate overfitting and/or confounding.

### Missing values

Missing values are common in MS data and are a key challenge in quantitative proteomics. Missing values arise when, for example, a peptide is identified in some samples but not in others; for the samples in which the peptide was not identified, abundances are not assigned or are assigned NA (not a number). A peptide may be missing because it is not present in the sample, it may be present but at a concentration below instrument detection limit, or may be present and not identified correctly or detected due to some unknown effect. Generally one cannot easily distinguish why peptide abundance is missing. What we do with the missing values, on the other hand, should ideally rely on the mechanism that caused the values to be missing. For example, we can usually separate missing values into two categories: missing completely at random (MCAR) and abundance-dependent missing values. MCAR values occur due to some "glitches" in the instrumentation, such as poor ionization, other peptides competing for charge, *etc*. This means that the fact that a peptide was unobserved in a sample has nothing to do with its abundance or the abundances of other peptides. Such peptide measurements can be described as missing in an "abundance-independent" manner. Random missingness is expected to affect a relatively small proportion of the peptides [22]. Meanwhile, "abundance-dependent" missingness in MS data boils down to censoring. In this case, a peptide abundance falls below the instrument detection limit or a peptide is simply not present. Knowing that a peptide is censored gives us partial information about the peptide intensity, in that we know it must be less than the detection limit of the instrument. This is an example of left-censoring. Right-censoring would occur if the detector were to get saturated with signal and fail to record abundances above a certain threshold. Right-censoring is less-commonly observed in current MS-based proteomics.

Values missing completely at random (MCAR) can be imputed by simple methods, although some methods are better than others [12]. For example, imputing MCAR values with row means or lowest observed value is computationally very fast and easy to implement, but imputing multiple values with the same value will underestimate the true biological variation as the number of imputed values increases. On the other hand, given a reasonable number of observations for a peptide, MCAR values can be imputed from an empirical probability distribution. For instance, log-transformed intensities across samples are approximately Normally distributed, making it possible to impute MCAR measurements from the Normal distribution with mean and variance estimated from the observed data. The values imputed in such a way will vary and thus variance underestimation will not be as large an issue. Missing values from different disease groups may need to be imputed from distinct distributions, as means of those disease groups may be different. Imputation of MCAR and effects of the imputation method on subsequent inference has been heavily studied in microarray data and most methods used in proteomics are inherited from microarray analysis [23].

Censored data present a more complicated problem, as observed values are not a good basis for imputation. In this scenario, censored values are said to be informative, in that the fact that a peptide was not observed tells us that its abundance was simply below our ability to detect it. The observed values for a peptide are not representative of the unobserved values, and analyzing only the observed values or performing imputation based on their average, or even random values generated from an estimated probability distribution as described above, will result in upward-biased estimates and downward-biased standard errors.

**Figure 1:**
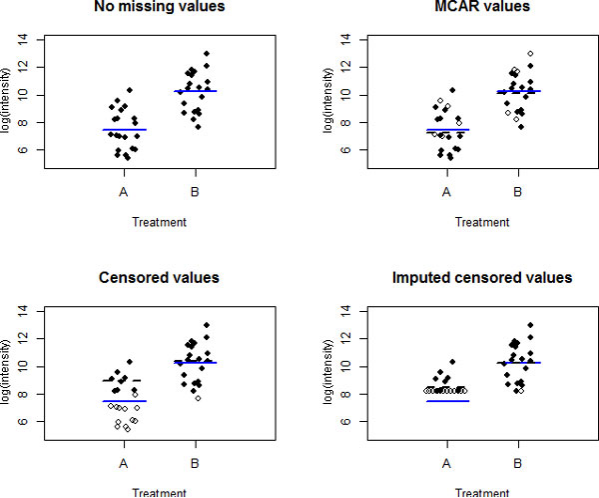
**Examples of missing data**. Intensities for a peptide with two treatment groups with (A) no missing values, (B) MCAR missing values, (C) censored missing values, and (D) censored missing values imputed as a minimum observed value.

**Figure 2:**
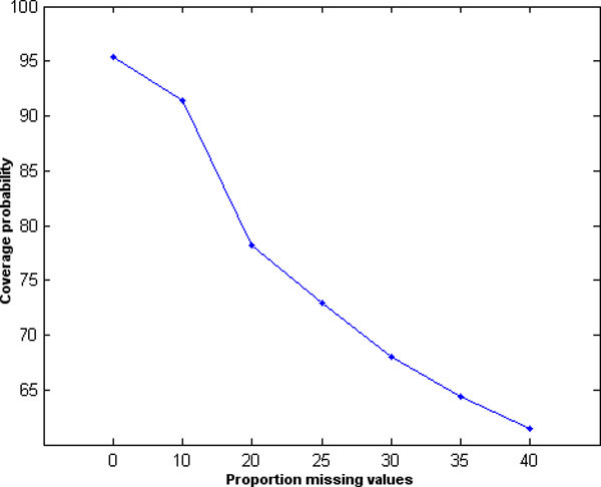
**Percent coverage for nominal 95% confidence intervals of protein-level differences**.

Figure 1 shows peptide intensities for two treatment groups with (A) no missing values, (B) MCAR missing values, (C) censored missing values, and (D) censored missing values imputed as a minimum observed value. Blue lines represent the true group mean and black dashed lines represent the mean estimated with missing or imputed values. Importantly, it is hard to tell in practice whether a missing observation is censored or MCAR; a proper probability model can help [22,24]. Figure 2 shows actual coverage proportions for nominal 95% confidence intervals of protein-level differences, based on 100 simulations of 200 proteins with varying numbers of peptides. Percent missing values was varied from 0% to 40% with 5% missing MCAR values in all but the 0% missing dataset. Whereas we expect 95% of all 95% confidence intervals to contain their true parameter value, actual coverage decreases sharply as the number of censored values increases.

**Figure 3:**
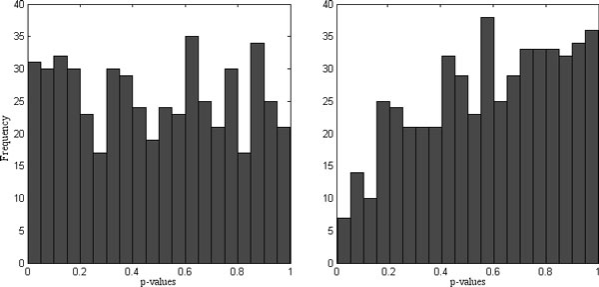
**Histograms of the null p-values for normalized (left) and raw (right) peptide abundances**.

In prior work, we proposed a statistical framework for protein quantitation and inference in the presence of values missing completely at random as well as censored [22]. Our model for protein-level abundance is similar to that in Equation 1:

(2) $$y_{ijkl}=\text{Pro}{\text{t}}_{i}+\text{Pe}{\text{p}}_{ij}+\text{Trea}{\text{t}}_{ik}+\varepsilon_{ijkl}$$

A maximum likelihood model is employed that expresses protein-level abundances in terms of peptide-level abundances and accounts for the two types or missingness. Statistical inference proceeds by numerically optimizing the maximum likelihood model to obtain p-values for differential protein expression. It is also possible to use rough parameter estimates to perform model-based filtering and imputation without the use of numerical optimization to obtain imputed values for future inference. Note that model (2) represents one approach for 'rolling up' peptide-level information to the protein level. In general, peptide-to-protein rollup is a complex exercise, and others have taken a variety of approaches [25,26].

In the automated filtering routine, information content from maximum likelihood theory guides the selection and exclusion of peptides and proteins. Model (2) is constructed under the assumption that primary interest is in protein-level group differences; this would make the Treat*_ik _*the parameters of interest. For any protein and a set of peptides within that protein, the Fischer information matrix is estimated and information content is quantified for the protein-level group difference parameters by taking the scaled determinant of the corresponding matrix block. Proteins are filtered if no collection of peptides produces an identifiable model, with non-zero information matrix determinant. A greedy search algorithm is then used to select a subset of peptides for each remaining protein that produce an optimal information content. The remaining peptides are filtered out. Further, an imputation routine generates values for missing observations from a normal distribution. The values are generated from a left hand tail of the distribution (below a censoring cutoff) if an observation is determined to be censored and from the complete normal distribution if it is determined to be MCAR. A single imputation is carried out and p-values are adjusted to minimize the effect of possible overfitting. P-value adjustment relies on the assumption that null p-values are uniformly distributed and thus the histogram of p-values should look flat with a spike at the small p-values; these are differentially expressed proteins. A deviation from such a picture may be indicative of overfitting (in the non-normalized p-value histogram, it may indicate bias). Adjustment is performed to assure that null p-values are approximately uniformly distributed. Since in biological samples it is not known which proteins are differentially expressed and which ones are not, only the right tail of the p-value distribution is considered as true null p-values in the adjustment. The algorithm is implemented in the DanteR statistical software toolbox available at: http://omics.pnl.gov/software/ and from the author's website.

Luo et al. 2009 proposed a Bayesian approach to dealing with censored (they call them non-randomly missing) observations in iTRAQ (isobaric Tags for Relative and Absolute Quantitation) data. The authors use logistic regression to determine if a peptide is censored or MCAR. This is based on the assumption that there is a negative correlation between probability of missing value and peptide abundance and an approximate linear relationship between the missing peptide probability and the observed intensity at the logit scale. Logistic regression is a nice fit for a problem where a distinction needs to be made between only two classes, here they are censored and MCAR values. Although the authors apply their model to labeled data, it can also be applied to unlabeled MS data. The authors perform inference while taking into account censored missing values but no model for actual imputation of missing values is proposed. In this sense, using the proposed approach is similar to using the maximum likelihood model (Karpievitch et al. 2009b) to obtain the p-values for differential expression; we can get the p-values but not the p-values for imputed data. No implementation has been made available to the public as described in the manuscript.

The choice of thresholds to use when identifying peptides is related to the problem of missing values, although the specific nature of this relationship is not known. Lowering the amount of evidence required for identifying peptides (lowering the threshold) will result in more peptides and peak intensities but will not necessarily result in a decrease in the number of missing values. In fact, it might be expected that lower identification thresholds will lead to a greater number of missing values, since a greater proportion of the additional peptides are liable to be false identifications. Having said that, a careful examination of these issues would make for interesting future work.

Missing values may also occur due to the limited depth of coverage of the instruments. In MS/MS identification, generally only a small portion of the most abundant peptides at a given time (MS^1^) are selected for further fragmentation and identification in the MS^2 ^phase. Thus, if a peptide is of lower abundance in one treatment group vs. the other it may not be identified if there are enough more-abundant peptides in the same MS scan. This issue can be addressed by selecting specific masses for further fragmentation instead of the top most abundant peptides.

### Impact of complex preprocessing on downstream statistical inference

Normalization and missing value imputation generally occur as preprocessing steps followed by statistical inference to answer questions of primary scientific interest. However, all data processing, including both preprocessing and downstream inference, "uses up" some of the information in the data to fit and employ statistical or mathematical models. Ideally, a single statistical model would be used to simultaneously carry out preprocessing and inference [18]. We might represent this model as such:

(3) Data=Technical Signal+Biological Signal+Random Error

where Technical Signal represents any systematic biases as well as missing-data patterns, and Biological Signal represents systematic biological differences between comparison groups of interest.

The most natural approach to data analysis based on the above model would be to fit it in a single step. This would correspond to carrying out preprocessing and inference simultaneously. In practice, the typical analysis pipeline, composed of preprocessing steps followed by downstream inference, is analogous to first fitting the model:

(4) Data=Technical Signal+Random Error

then carrying out inference on the basis of the model:

(5) Residuals=Biological Signal+Random Error

where Residuals are the processed (normalized) data. The problem with this approach is that the variability introduced into the pipeline from the first step is not communicated to the second step. In other words, any degrees of freedom that are used up in the preprocessing model are not discounted when using the second model for inference. This can lead to an "overfitting" of the data, whereby resulting statistical inferences are not directly interpretable [27].

As mentioned above, to minimize the issues of overfitting, inference and normalization can be carried out simultaneously [18,21]. If that is not possible, in cases when normalized data needs to be passed over to investigators, additional variables can be provided that should be used in further analysis together with the normalized data [17]. Such variables can be stored and plugged-in as covariates in the significance-testing model, for example, added to the regression model matrix. Carry-over variable are not always feasible and there is no guarantee that the collaborators will actually use them in the future significance analysis. To avoid the requirement for carry-over variables, methods that adjust for possible overfitting should be applied to the normalized data [8]. This approach adds random variability to the model residuals to effectively remove the appropriate number of degrees of freedom. This allows for post-normalization significance analysis with no special steps required; that is, no additional covariates need to be added to the inference model, and no adjustment to the null sampling distribution of test statistics are required.

### Combining normalization and missing value imputation

At this point we have shown that systematic bias and missing observations are prevalent in MS-based data. The fact that many normalization routines require a 'complete' matrix with no missing values, raises a question: should the imputation be done first followed by normalization? That seems like a reasonable solution at first. For example, one can impute missing values using one of the methods described above to produce a complete matrix, and then use one of the normalization routines to remove bias. At this time one should wonder if the imputation was to be repeated would the values be different and would that affect normalization? The answer is generally, yes, imputed values will be different every time because they are drawn at random from an appropriate distribution. Thus, imputed values, especially if there is a high proportion of those, can potentially obscure the bias trends and prevent normalization routines from effectively removing it. Moreover, it does not make sense to impute missing values based on biased observations.

We show the impact that normalization and imputation and the order of those procedures may have on the processed data and significance analysis by using simulated data. Simulated data were created with 10 samples in each of two treatment groups (20 samples total). The size and structure of the simulated data were selected to mimic those in a real dataset composed of human subjects. Specifically, 1400 proteins were simulated with varying numbers of peptides per protein and 13% of missing values. Proportion MCAR values was simulated to be 5% and 8% censored values. Simulated data were generated from model (6), which is an adaptation of model (1) that (through the Samp*_im _*terms) allows for sample-specific variation in the peptide abundances, such as might be induced by differences in the loading amounts from sample to sample:

(6) $$y_{ijkl}=\text{Pro}{\text{t}}_{i}+\text{Pe}{\text{p}}_{ij}+\text{Trea}{\text{t}}_{ik}+\text{Sam}{\text{p}}_{im}+\varepsilon_{ijkl}$$

**Figure 4:**
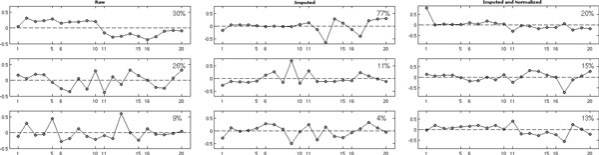
**Top three eigentrends identified in raw (left), imputed (middle); and normalized after imputation data (right)**. X-axis is the sample index, y-axis are values in eigentrends.

The index *m *is for sample, and the remaining model terms are as in model (1). We perform (i) imputation followed by normalization and (ii) normalization followed by imputation. We use normalization and imputation methods described in Karpievitch et al. 2009a and 2009b, but any other appropriate methods will have similar effects. Figure 4 shows the eigentrends in raw (top left), residual (top right), imputed (bottom left), imputed and normalized (normalized and imputed) data; the top three trends are shown for each subplot. Eigentrends are trends identified in the data by using SVD, and they capture major variations in the data. In the differential expression (DE) studies one would ideally like to see a step function for each group, reflecting mean differences between groups. In this scenario, where imputation is done first followed by normalization, the randomness introduced by the imputation process covers up the true biological trends as well as bias. Figure 4 shows that in raw data, 30% of the variation is attributable to the differences between the groups (left panel). After imputation, the DE trend (trend that looks like a step) is no longer detectable (Figure 4, middle panel). This suggests that bias (second trend in the raw data that explains 26% of variation) does not allow us to perform imputation correctly. Normalization performed after the imputation does not seem to be able to remove bias well enough to discern the group differences (Figure 4, right panel), suggesting that this approach is not appropriate.

**Figure 5:**
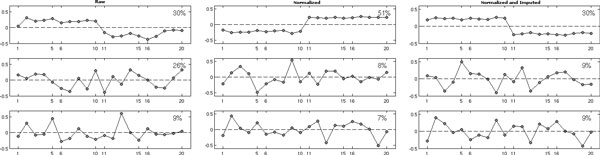
**Top three eigentrends identified in raw (left), normalized (middle); and imputed after normalization data (right)**. X-axis is the sample index, y-axis are values in eigentrends.

Normalization followed by imputation, on the other hand, performs better (Figure 5). First, the DE trend (trend that looks like a step) after normalization is more apparent and explains a higher percentage of variation (Figure 5, left panel). Second, the DE trend remains the most significant trend after imputation (Figure 5, right panel). It is sensible for the percent variation explained to go down after imputation, as more data is available for computing the trends. As we saw in Figure 4, imputed values are still random draws from an appropriate distribution, and they allow for more peptides and variation to be added to the data. Eigentrends are computed only from the peptides with no missing values, thus a smaller pull of peptides is used in raw data and in data normalized before imputation. We suggest that normalization be done first followed by missing value imputations. Software tools such as DanteR as well as stand-alone functions in R and Matlab may be used to perform normalization, imputation, significance analysis and visualization.

## Discussion

Quantitative proteomic data present complex challenges to the data analyst. We have discussed two common issues in the context of spectral peak intensity analysis, involving biases due to systematic technical variation and informative missingness patterns. Normalization is the solution to biases, but the normalization techniques employed must be simultaneously flexible enough to capture arbitrary patterns and delicate enough to not overfit the data. Importantly, some of the biases such as sample/subject selection bias may not be corrected by a normalization technique as in some cases subjects are selected not entirely at random, such as subjects visiting a doctor about a specific condition. Missing values, meanwhile, greatly complicate the statistical analysis of quantitative proteomic data, particularly as missingness in this context is largely synonymous with censoring. However, standard statistical techniques can be used to facilitate valid statistical conclusions.

## Competing interests

The authors declare that they have no competing interests.

## Authors' contributions

YVK, ARD, and RDS contributed equally to the general formulation and layout of the paper. YVK led the writing and revision.
